# A whole-plant perspective of isohydry: stem-level support for leaf-level plant water regulation

**DOI:** 10.1093/treephys/tpab011

**Published:** 2021-02-16

**Authors:** Henrik Hartmann, Roman Mathias Link, Bernhard Schuldt

**Affiliations:** Department of Biogeochemical Processes, Max Planck Institute for Biogeochemistry, Hans-Knöll Str. 10, 07745 Jena, Germany; Julius-von-Sachs-Institute of Biological Sciences, Ecophysiology and Vegetation Ecology, University of Würzburg, Julius-von-Sachs-Platz 3, 97082 Würzburg, Germany; Julius-von-Sachs-Institute of Biological Sciences, Ecophysiology and Vegetation Ecology, University of Würzburg, Julius-von-Sachs-Platz 3, 97082 Würzburg, Germany

**Keywords:** capacitance, hydraulic segmentation, leaf deciduousness, leaf water potential, non-structural carbohydrates, osmoregulation, osmotic adjustment, stomatal control, turgor loss point, xylem embolism resistance

The woody vegetation, in particular trees, is facing difficult times in many regions across the globe. Unprecedented rapid increases in average temperatures along with increasingly frequent periods of extreme drought and heat outrun the acclimatory capacities of many tree species and other woody plants. Observations of increased tree mortality and severe forest decline are accumulating in all forested biomes (Allen et al. 2010, Hartmann et al. 2018) and questions arise as to whether and how the woody vegetation may be able to cope with these changes (Allen et al. 2015) and which species will succumb to drought while others survive.

More than a decade ago, the seminal paper by McDowell et al. (2008) addressed this question with the ‘hydraulic framework’, linking tree mortality mechanisms to the stomatal behavior of a tree species and to duration and severity of a drought. It was speculated that species that close stomates early during drought become dependent on stored carbohydrates as photosynthetic rates and sugar production decline along with the decline in gas exchange. In contrast, species that maintain high photosynthetic rates by keeping stomates open longer during drought risk strains on the conducting tissue as the decreasing water potential induces high tensions in the xylem, which might lead to the formation of embolisms and their propagation to adjacent conduits.

The regulation of water potential has been described in terms of plant control of stomatal conductance (Tardieu and Simonneau 1998). While more isohydric species maintain leaf water potential (*P*_L_) relatively constant during changing environmental conditions (soil and atmospheric drought), more anisohydric species let *P*_L_ covary congruently with environmental fluctuations (Martínez-Vilalta and Garcia-Forner 2017) until the water potential at stomatal closure (*P*_st_) is reached (Martin-StPaul et al. 2017). In perfectly anisohydric species, predawn water potential (*P*_pd_) thus corresponds to the soil water potential in the soil horizon where water is taken up from (Plaut et al. 2012). Ultimately, when the soil–root interface is disconnected (Carminati and Javaux 2020), *P*_pd_ should approximate *P*_st_. According to the above-mentioned framework, isohydric species would be more prone to carbon starvation, while anisohydric species are more likely to die from tissue desiccation via hydraulic failure.

The iso/anisohydry concept was introduced by Berger-Landefeldt (1936) to classify species groups that differed in their diurnal transpirational behavior and thus leaf water content under non-drought conditions. His concept evolved from Walter’s (1931) definition of thresholds for the water status regulation of different plant types according to their osmotic potential into stenohydric (small fluctuation in water status, hydro-stable) and euryhydric (large fluctuation in water status, hydro-labile) species. Ever since, attempts to categorize species according to their water relations strategy into isohydric or anisohydric have shown modest success (see Hochberg et al. 2018 and references therein). The regulation of plant water status across the continuum of stomatal regulation is highly complex, and neither Walter (1931) nor Berger-Landefeldt (1936) were able to report a sharp distinction between the two groups. Notwithstanding, many subsequent studies referred to an ambiguous dichotomy between extremes along this continuum (cf., Martínez-Vilalta et al. 2014). The apparent simplicity of the concept may have led to misinterpreting isohydry as a simple functional trait or a strategy defined by the isolated action of stomates and not a response of the entire plant in order to regulate the water status (Hochberg et al. 2018). The work by Jiang et al. breaks this boundary and offers a whole-plant perspective.

In their article, Jiang et al. investigate the regulation of water status during the dry season in 24 woody species distributed along a gradient of isohydry. For the first time, this study focuses on how this regulation relates to stem capacitance and non-structural carbohydrate (NSC) storage, linking leaf-level responses to whole-plant regulation. The main finding is that there is a trade-off between stem capacitance and stem NSC storage, where species that make greater use of stem water storage have a smaller NSC storage pool. Less isohydric water potential regulation is associated with greater NSC storage depletion during the dry season, most likely as a consequence of osmotic adjustment. More isohydric species, by contrast, are characterized by greater stem water storage use. Surprisingly, and contrary to what the hydraulic framework would predict, more isohydric species showed an accumulation of NSC during the dry season, apparently because the use of stem water allowed earlier leaf flushing and higher photosynthetic rates. As such, the contribution by Jiang et al. provides an additional dimension to research on water regulation and drought responses and suggests a mechanistic explanation for why NSC storage pools often—but not always—decline during drought (Adams et al. 2017). These contrasting observations on NSC depletion during drought are likely a result of differences in the species’ stomatal control strategy. Most likely, only species that follow a more anisohydric stomatal control strategy rely on the conversion of starch into osmotically active sugars in order to lower the osmotic potential of living cells, in both leaves and stems.

**Figure 1.**
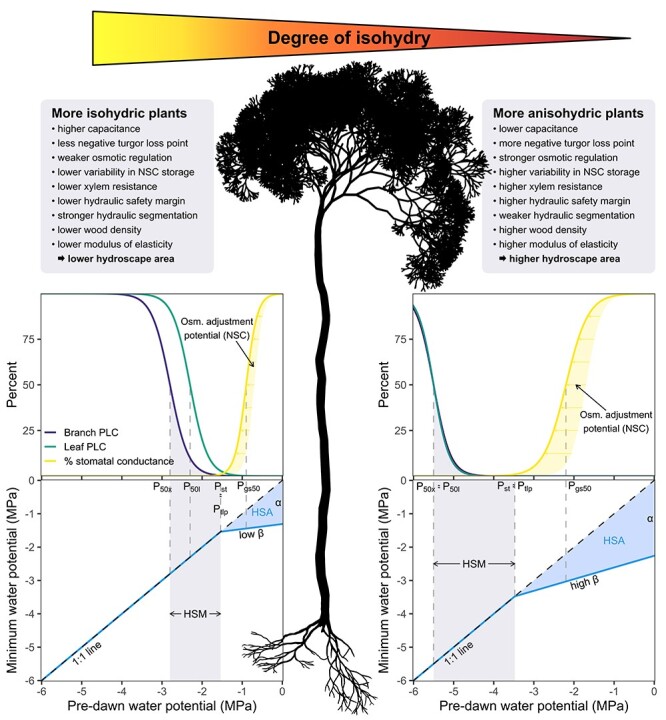
Top: Whole-plant traits commonly associated with iso/anisohydry. Bottom: Conceptual diagrams for two hypothetical species from the two ends of the continuum of isohydry (left: more isohydric; right: more anisohydric). Adapted and modified from Meinzer and McCulloh (2013) and Charrier et al. (2018). The upper panels of the inset figures show branch and leaf vulnerability curves (percent loss of branch/leaf conductivity vs xylem pressure) as well as stomatal response curves (reduction of stomatal conductance with increasingly negative water potential; with yellow arrows indicating the potential of NSC-driven osmotic adjustment). The lower panels show the relationship between minimum and pre-dawn water potential and the corresponding HSA. Parameters used in the figure: Isohydric—xylem pressure at 50% loss of xylem (P_50×_) and leaf (*P*_50l_) conductance: −2.80 and −2.30 MPa, respectively; point of 50% stomatal closure (*P*_gs50_): −0.90 MPa; point of stomatal closure (*P*_st_): −1.54 MPa; hydraulic safety margin (HSM = *P*_st_ − *P*_50x_): 1.26 MPa; slope of the relationship between minimum and pre-dawn water potential (*β*): 0.15 MPa MPa^−1^; corresponding intercept (*α*): −1.31 MPa; HSA: 1.00 MPa^2^. Anisohydric—*P*_50×_: −5.50 MPa; *P*_50l_: −5.50 MPa; *P*_gs50_: −2.20 MPa; *P*_st_: −3.48 MPa; HSM: 1.26 MPa; *β*: 0.35 MPa MPa^−1^; *α*: −2.26 MPa; HSA: 3.93 MPa^2^, PLC, percent loss of conductivity.

A close coordination between stem and/or whole-plant hydraulic traits and stomatal control has been found repeatedly (Martin-StPaul et al. 2017, McCulloh et al. 2019, Pivovaroff et al. 2018). More isohydric species are often characterized by a relatively vulnerable xylem and light wood, and must rely on a high stem capacitance (amount of released stored water per unit change in water potential) as well as low modulus of leaf elasticity (increased leaf cell wall elasticity) to maintain gas exchange until *P*_st_ is reached (Plaut et al. 2012, Meinzer and McCulloh 2013, Leuschner et al. 2019, Jiang et al. 2020b). Due to hydraulic vulnerability segmentation between leaf petioles and stem xylem (Losso et al. 2019), drought-induced premature leaf senescence occurs as a protective measure in these species (Figure 1). Osmotic adjustment, on the other hand, seems more important in more anisohydric species, resulting in a larger plasticity in the water potential at turgor loss point (*P*_tlp_). The maintenance of water loss via declining *P*_L_ is facilitated by this biochemical mechanism that increases the concentration of osmotically active substances to lower the osmotic potential for preventing turgor loss in living plant cells (Blum 1958, Kozlowski and Pallardy 2002, Sanders and Arndt 2012). The larger potential for osmotic adjustment in more anisohydric plants permits them to lower their *P*_tlp_ over the course of the growing season and thus to keep their stomates open and maintain photosynthetic activity at more negative water potentials (Figure 1).

Despite ongoing controversies about the usefulness of the iso/anisohydry concept (Klein 2014, Martínez-Vilalta and Garcia-Forner 2017, Hochberg et al. 2018, Fu and Meinzer 2019, Ratzmann et al. 2019), it appears still useful for describing hydraulic behavior of plants as it integrates several relevant components of whole-plant responses to drought. While a unique definition of isohydry is still missing (Martínez-Vilalta and Garcia-Forner 2017), the recently introduced concept of the ‘hydroscape area’ (HSA) (Meinzer et al. 2016) represents a major step in that direction (cf., Li et al. 2019). Building upon the framework by Martínez-Vilalta et al. (2014), the HSA defines the water potential landscape, i.e., the ranges of predawn and midday potentials, over which stomata are able to regulate leaf water potential during soil drying and prior to drought-induced stomatal closure (Meinzer et al. 2016, Fu and Meinzer 2019). In the hydroscape framework, the degree of isohydry (i.e., the degree of stringency in the stomatal response) of a plant is expressed based on the area between the 1:1 line and the regression through midday (*P*_md_) versus pre-dawn (*P*_pd_) leaf water potential (blue triangular area in Figure 1). As anisohydric species under dry conditions tend to close their stomates later than isohydric species, their *P*_md_ is below their *P*_pd_ over a wider range of water potentials, which results in a larger HSA. A low slope β in this relationship (Fu and Meinzer 2019), for example, is indicative of a reliance on internally stored water, while high values of β suggest an influence of osmotic adjustment (Figure 1). Notably, as *P*_md_ has to approach *P*_pd_ after complete stomatal closure (Meinzer et al. 2016), which therefore dictates the position of one of the points limiting the HSA, changes in *P*_st_ driven by osmotic adjustment may result in certain degree of plasticity in this trait (Figure 1). Furthermore, the direct link between the HSA and *P*_st_ indicates that it may be useful as a complement to hydraulic safety margins based on stomatal response (cf., Martin-StPaul et al. 2017), as they form a pair of composite variables describing plant drought responses before and after reaching the point of stomatal closure.

The study by Jiang et al. highlights the importance of a holistic whole-tree approach that acknowledges the interdependency of the water and carbon balance of trees. As identified by Martínez-Vilalta and Garcia-Forner (2017), isohydric species might not be more carbon limited than anisohydric species, in contrast to the framework proposed by McDowell et al. (2008). Instead, the conversion of starch to osmotically active substances seems more important in more anisohydric species for shifting the *P*_tlp_ in response to seasonally declining *P*_L_. Future research should assess the role of starch conversion in response to abscisic acid signals (Thalmann and Santelia 2017) as a regulatory mechanism to lower the osmotic potential in living cells. Several further interesting questions arise from the study by Jiang et al. (i) Is osmotic adjustment only important for more anisohydric species, and at what water potential does the conversion from starch to osmotically active sugars begin? (ii) What happens with these sugars after returning to a normal water status and are they still available for other biochemical processes? (iii) During drought, how much carbon is used for osmotic adjustment and how much is available for respiration? (iv) Does osmotic adjustment of the stomatal response confer a link between NSC status and HSA?

Answering such questions would also feed back into the hydraulic framework of McDowell et al. (2008), as both water and carbon relations become intertwined to a point where carbon starvation does not merely refer to a lack of substrates for maintenance respiration, but a supply shortness for the ensemble of plant functional processes, in particular during drought. Measurements of carbohydrate concentrations thus lose their ability to track the plant catabolic reservoir. Ultimately, to fully understand the causal relationships between carbon metabolism and stomatal regulation as well as plant water relations, it is imperative to study their behavior in response to the direct experimental manipulation of NSC concentrations (cf., Sapes et al. 2020).
